# Oral *Panax notoginseng* Preparation for Coronary Heart Disease: A Systematic Review of Randomized Controlled Trials

**DOI:** 10.1155/2013/940125

**Published:** 2013-08-20

**Authors:** Qinghua Shang, Hao Xu, Zhaolan Liu, Keji Chen, Jianping Liu

**Affiliations:** ^1^Beijing University of Chinese Medicine, Beijing 100029, China; ^2^Xiyuan Hospital, China Academy of Chinese Medical Sciences, Beijing 100091, China; ^3^Centre for Evidence-Based Chinese Medicine, Beijing University of Chinese Medicine, Beijing, China

## Abstract

This systematic review aims to evaluate current evidence for the benefit and side effect of oral *Panax notoginseng* preparation for coronary heart disease (CHD). We included 17 randomized clinical trials (17 papers and 1747 participants). Comparing with no intervention on the basis of conventional therapy, oral *Panax notoginseng* did not show significant effect on reducing cardiovascular events, but it could alleviate angina pectoris (including improving the symptoms of angina pectoris [RR 1.20; 95% CI 1.12 to 1.28; 7 trials, *n* = 791], improving electrocardiogram [RR 1.35; 95% CI 1.19 to 1.53; 8 trials, *n* = 727], decreasing the recurrence of angina pectoris [RR 0.38; 95% CI 0.16 to 0.94; 1 trials, *n* = 60], duration of angina pectoris [RR −1.88; 95% CI −2.08 to −1.69; 2 trials, *n* = 292], and dosage of nitroglycerin [MD −1.13; 95% CI −1.70 to −0.56; 2 trials, *n* = 212]); oral *Panax notoginseng* had no significant difference compared with isosorbide dinitrate on immediate effect for angina pectoris [RR 0.96; 95% CI 0.81 to 1.15; 1 trial, *n* = 80]. In conclusion, oral *Panax notoginseng* preparation could relieve angina pectoris related symptoms. However, the small sample size and potential bias of most trials influence the convincingness of this conclusion. More rigorous trials with high quality are needed to give high level of evidence, especially for the potential benefit of cardiovascular events.

## 1. Introduction

Coronary heart disease (CHD) is one of the most leading causes of morbidity and mortality in many countries with large economic and human burdens, and it accounts for 20% of overall mortality in the United State [1]. It is reported that Ischaemic heart disease is the second leading cause for males and the third leading cause of global burden of disease for females, accounting for 6.8% and 5.3% respectively [2]. Although the benefit of some conventional drugs, such as aspirin and statin, have been demonstrated in reducing CHD mortality, annually 17.3 million people die from cardiovascular disease (CVD) worldwide (WHO 2008), and over 80% of CVD deaths take place in low and middle income countries, it is reported that by 2030 more than 23 million people will die annually from CVDs [3].

In recent years, traditional medicines have been playing more and more important roles in the maintenance of health, the prevention and treatment of diseases, and plant-based drug discovery [4–8]. Chinese herbal medicine or its products have been administered widely for treating CHD in China. There are more than one hundred kinds of patent herbal medicine for CHD available at present. Puerarin injection [9], Danshen preparations [10], Tongxinluo [11], compound salvia pellet [12], Suxiao jiuxin wan [13] or traditional Chinese herbal products [14] have been shown as potential benefits recently by systematic reviews. Sanqi is one of the most widely used herbal medicines in China, with function of invigorating the blood circulation according to TCM theory. *Panax notoginseng* was the active and effective component purified from sanqi. Oral *Panax notoginseng* products included xuesaitong capsule, xuesaitong dripping pills, xuesaitong pill, xuesaitong effervescent tablet, xuesaitong granule, xuesaitong dispersible tablet, sanqishutong capsule, *Panax notoginseng* saponins (PNS) tablet and PNS capsule. The content of *Panax notoginseng* varies in different agents. All of the agents have been used in clinic for patients with CHD for decades of years. Recent researches found its antioxidative [15], antiatherogenic, lipid-lowering, and anti-inflammatory [16] effects and angiogenic effect [17]. A Cochrane systematic review indicated that *Panax notoginseng* was effective in preventing stroke [18]. Some recent clinical trials also proved that it could benefit CHD patients [19, 20]. Therefore, this systematic review aims to evaluate the safety and effectiveness of oral *Panax notoginseng* preparations for CHD patients.

## 2. Method

### 2.1. Inclusion Criteria

We included randomized controlled trials (RCTs) or cross-over trials in English and Chinese regardless of publication type in this review. Quasirandomized trials were excluded and the first stage of data was used if it was cross-over trial. Any adult participant with CHD meeting with at least one of the current or past definitions or guidelines of CHD (including acute coronary syndrome (ACS) and X syndrome) was considered. Those who did not introduce diagnostic criteria in the text but stated patients with definite CHD were also included. The trial was included if oral *Panax notoginseng* preparation was in intervention group regardless of dosage, treatment course, and agents; trials should be excluded if there were other Chinese herbal medicines in intervention group; trials also should be excluded if there was a combination of *Panax notoginseng* preparation and a kind of western medicine on the basis of control group. Chinese herbal injection should be excluded in this review. Placebo, no intervention, or nitrate was considered in control group, Chinese herbal medicine in control group should be excluded. Oral *Panax notoginseng* preparations versus conventional therapy (except for nitroglycerin) were excluded for limited extension. 

Outcome measures include primary outcomes: all cause mortality, cardiovascular events (e.g., CHD mortality, incidence of myocardial infarction (MI), revascularization, and rehospitalization for unstable angina); secondary outcomes: quality of life, attack of angina pectoris (measuring by recurrence of angina pectoris, frequency of angina pectoris, duration of angina pectoris, dosage of nitroglycerin, decrement of nitroglycerin, efficacy of angina pectoris, and others), electrocardiogram (ECG), and adverse events. We defined the efficacy of angina pectoris as improvement was more than 50%; the efficacy of ECG as elevation of ST segment was more than 0.05 mv.

### 2.2. Search Strategy

Two review authors (Qinghua Shang, Hao Xu) searched the following databases up to January 2013 independently for the identifications of trials (publication or nonpublication): the Cochrane Library, Pubmed, Chinese Biomedical database (CBM), China National Knowledge Infrastructure (CNKI), Chinese VIP Information (VIP), and Wanfang databases. We used the terms as follows: coronary heart disease, CHD, coronary artery disease, angina pectoris, myocardial infarction, acute coronary syndrome, cardi*, sanqi, sanchi, jinbuhuan, tiansanqi, tianqi, panlongqi, tongpitiegu, xueshancao, liuyuelin, xuesaitong, xueshuantong, notoginseng, pseudoginseng, *Panax notoginseng*, ginsenosides *Panax*, sanchinoside, and so forth. Because of different characteristics of various databases, MeSH terms and free text terms were used regardless of the report types in full text, title, keyword, subject terms, or abstract.

### 2.3. Data Extraction and Quality Assessment

Two review authors (Qinghua Shang, Hao Xu) independently extracted data according to a data extraction form made by the authors. Disagreements were resolved by consensus or consultation from a third reviewer (Jianping Liu or Zhaolan Liu). The methodological quality of trials was assessed independently using criteria from the Cochrane Handbook for Systematic Review of Interventions, Version 5.0.1 (Qinghua Shang, Hao Xu) [15]. The items included random sequence generation (selection bias), allocation concealment (selection bias), blinding of participants and personnel (performance bias), blinding of outcome assessment (detection bias), incomplete outcome data (attrition bias), selective reporting (reporting bias), and other biases. We judged each item from three levels (“Yes” for a low of bias, “No” for a high risk of bias, and “Unclear” otherwise), and then we assessed the trials and categorized them into three levels: low risk of bias (all the items were in low risk of bias), high risk of bias (at least one item was in high risk of bias), and unclear risk of bias (otherwise). 

### 2.4. Data Synthesis

We used Revman 5.1 software provided by the Cochrane Collaboration for data analyses. Studies were stratified by the types of comparisons. We will express dichotomous data as risk ratio (RR) and its 95% confidence intervals (CI). Continuous outcome will be presented as mean difference (MD) and its 95% CI. Heterogeneity was recognized significant when *I* ^2^ ≥ 50%. Fixed effects model was used if there is no significant heterogeneity of the data; random effects model was used if significant heterogeneity existed (50% < *I* ^2^ < 85%). Sensitive analysis would be used if there was any heterogeneity (including differences of clinical characteristics among trials and the statistical heterogeneity); subgroup analysis would be used in patients prescribed Xuesaitong softy capsule. Publication bias was explored using a funnel plot.

## 3. Results

### 3.1. Description of Included Trials

**Figure 1:**
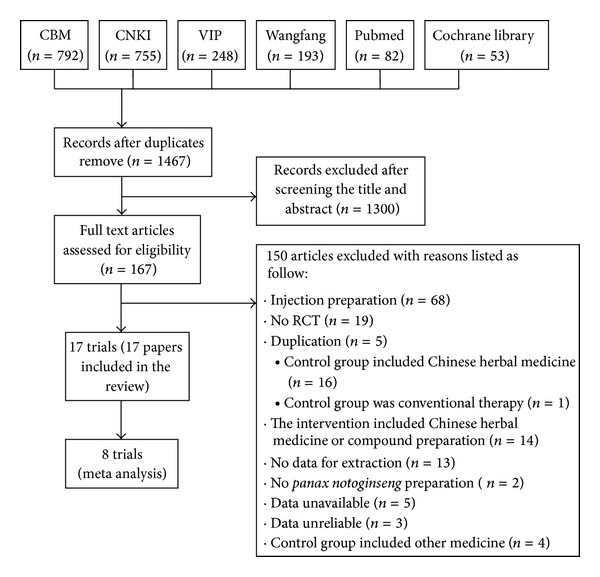
The process of included and excluded studies.

17 RCTs (17 papers) [19, 21–36] were included. All of the papers were published in Chinese and 2 were in postgraduate dissertations (unpublished study) [23, 24]. The whole process of trials selection was demonstrated in Figure 1. The characteristics of included trials were listed in Table 1.

1747 Participants were included (864 in the intervention group and 883 in the control group). 906 males and 581 females were included in 17 trials (two of the trials did not report the number in each gender group). A total of 7 criteria of CHD (including ACS) were involved. 5 trials [21, 27, 30, 33, 36] did not introduce criteria of CHD but mentioned that “patients with CHD were eligible to be included.” One trial [23] included patients who need to take percutaneous coronary intervention (PCI) the next day; 8 trials [19, 21, 22, 24, 27, 28, 31, 33] included patients with unstable angina; 2 trials [34, 35] included patients with stable angina pectoris; 1 trial [25] included patients with either stable angina or unstable angina; the other 5 trials [26, 29, 30, 32, 36] did not introduce the types of CHD, but two of them recruited hospitalized patients [29, 32].

Patients in 11 trials [19, 21–23, 26–29, 31, 33, 36] were prescribed Xuesaitong softy capsule 2 tablet (120 mg, 60 mg *Panax notoginseng* Saponins [PNS] in each capsule) BID (regulation was conducted for the course); patients in 2 trials [32, 35] were prescribed Xuesaitong softy capsule 2 tablet (120 mg, 60 mg PNS in each capsule) TID; patients in 1 trial [34] were prescribed Sanqi guanxinning pills 2–4 pills (100 mg PNS in each pill) TID; 1 trial [25] prescribed PNS tablets 2–4 pill (50 mg PNS in each pill) TID, oral administration or sublingual administration, 2 trials [26, 30] used sanqi power (the purity is unclear) in the treatment group. The treatment course of treatment ranged from 7 days to 6 months.

There were 2 comparisons in the review according to various control groups: (1) *Panax notoginseng* preparations and conventional therapy versus conventional therapy (15 trials) [19, 21–24, 26–29, 31–36]; (2) *Panax notoginseng* preparations and conventional therapy versus nitrates and conventional therapy (2 trials) [25, 30]. Two trials [24, 28] were designed as three groups and four groups, respectively. Wang et al. [28] designed three groups with 2 comparisons: *Panax notoginseng* preparations and conventional therapy versus conventional therapy; *Panax notoginseng* preparations and trimethazine and conventional therapy versus conventional therapy; however, we extracted the data of first comparison for inclusion criteria. Ji and Zhang [24] designed four groups with 3 comparisons: *Panax notoginseng* coarse power and conventional therapy versus conventional therapy; *Panax notoginseng* semi-micron power and conventional therapy versus conventional therapy; *Panax notoginseng* micron power and conventional therapy versus conventional therapy; however, we summed up the three groups included *Panax notoginseng* as intervention group and conventional therapy as control group for data analysis.

### 3.2. Methodological Quality of Included Trials

**Figure 2:**
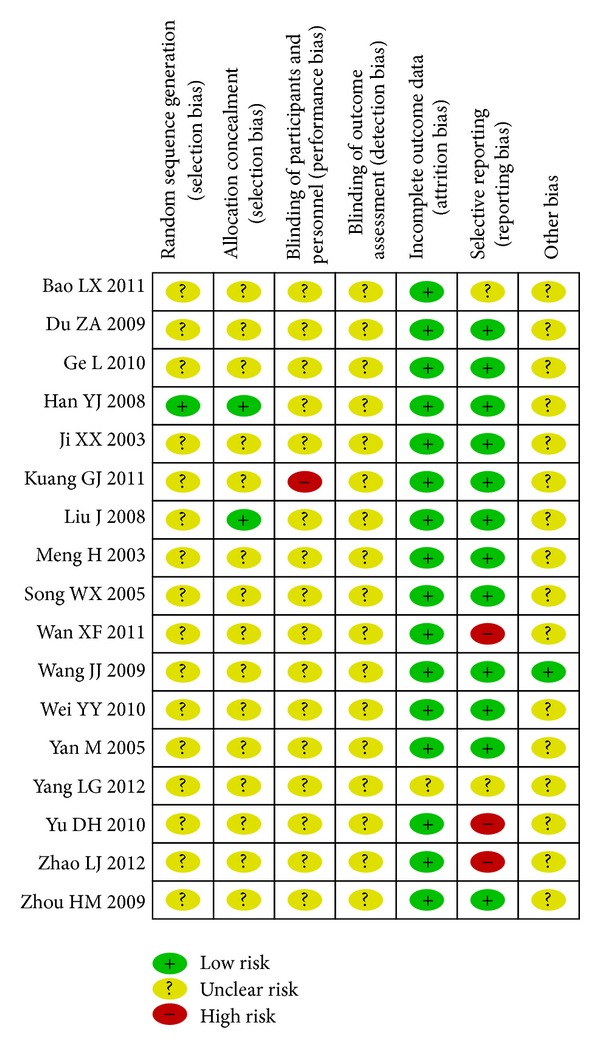
Risk of bias summary.

According to the criteria introduced above, no trial was evaluated as low risk of bias. Only one trial of the 17 trials reported the method to generate the allocation sequence (random number table) [23]. Two trials were assessed as having adequate concealment (concealed letter cover) [19, 23]. No trial reported blinding method. One trial [31] reported the result of followup. No trial reported information on withdrawal/dropout. All of trials provided baseline data for the comparability among groups. The results of the assessment of risk of bias are presented in a “risk of bias summary” figure produced by Revman 5.1 automatically (Figure 2).

### 3.3. Effect Estimates of Outcomes (Tables 2 and 3)

#### 3.3.1. Cardiovascular Mortality

There was only 1 trial [31] that reported the cardiovascular mortality in the comparisons of *Panax notoginseng* preparations (Xuesaitong softy capsule) and conventional therapy versus conventional therapy with no significant difference between the two groups [RR 0.50; 95% CI 0.05 to 5.34; 1 trial, *n* = 100]. In the followup of 4 months, 2 patients died of heart failure in the conventional therapy group and 1 patient died of arrhythmia in the combined therapy group. 

#### 3.3.2. Incidence of Myocardial Infarction (MI)

There were 2 studies [28, 31] reporting MI incidence in one comparison. Compared with no intervention on the basis of conventional therapy, *Panax notoginseng* preparations (Xuesaitong softy capsule) showed no significant reduction of incidence of MI (RR 0.17; 95% CI 0.02 to 1.37; 2 trials, *n* = 300) [28, 31]. 

#### 3.3.3. Incidence of Intractable Angina Pectoris

One trial [28] reported the intractable angina pectoris in 2 different comparisons. In the comparisons of *Panax notoginseng* preparation (Xuesaitong softy capsule) and conventional therapy versus conventional therapy, *Panax notoginseng* preparation (Xuesaitong softy capsule) showed no significant difference (RR 0.55; 95% CI 0.21 to 1.42; 1 trial, *n* = 200) in controlling intractable angina pectoris. 

#### 3.3.4. Rehospitalization for Unstable Angina

There was 1 trial [23] reporting rehospitalization. Compared with no treatment on the basis of conventional therapy, *Panax notoginseng* preparation (Xuesaitong softy capsule) showed no significant difference in the number of rehospitalization (RR 0.33; 95% CI 0.04 to 3.03; 1 trial, *n* = 60). 

#### 3.3.5. Recurrence of Angina Pectoris

One trial [23] reported recurrence of angina pectoris. Compared with no treatment on the basis of conventional therapy, *Panax notoginseng* preparation (Xuesaitong softy capsule) showed significant difference in reducing recurrence of angina pectoris (RR 0.38; 95% CI 0.16 to 0.94; 1 trial, *n* = 60). 

#### 3.3.6. Reduction of Nitroglycerin

The definition of successful nitroglycerin reduction was that the patients in the trial stopped using nitroglycerin or the dosage of nitroglycerin was cut off more than 50% after the trial. Two trials [24, 30] reported the condition of nitroglycerin. The results showed no significant improvement of *Panax notoginseng* preparation comparing with no treatment on the basis of conventional therapy (RR 1.41; 95% CI 0.89 to 2.24; 2 trials, *n* = 250).

#### 3.3.7. Angina Pectoris Alleviation

We defined the efficacy of angina pectoris as alleviation of more than 50%. There were 7 studies [22, 24, 27, 29, 31–33] reporting angina pectoris alleviation. The results showed significant improvement of *Panax notoginseng* preparations as compared with no treatment on the basis of conventional therapy (RR 1.20; 95% CI 1.12 to 1.28; 7 trials, *n* = 791). Subgroup analysis showed that Xuesaitong softy capsule in 6 trials [17, 23, 25, 27–29] was more effective than no treatment in the basis of conventional therapy (RR 1.19; 95% CI 1.11 to 1.27; 6 trials, *n* = 641).

#### 3.3.8. Electrocardiogram Improvement

We defined the efficacy of ECG as elevation of depressed ST segment of more than 0.05 mv. There were 9 trials [19, 22, 24–27, 31, 32, 35] reporting the electrocardiogram improvement. The results in 8 trials [19, 22, 24, 26, 27, 31, 32, 35] showed significant improvement of *Panax notoginseng* preparation comparing with no treatment on the basis of conventional therapy (RR 1.35; 95% CI 1.19 to 1.53; 8 trials, *n* = 727). 1 trial [25] showed that notoginsenoside pill had no immediate effect on improving ECG compared with isosorbide dinitrate (RR 0.79; 95% CI 0.41 to 1.52; 1 trial, *n* = 80). Subgroup analysis showed that Xuesaitong softy capsule [19, 22, 26, 27, 31, 32, 35] was superior to no treatment on the basis of conventional treatment in improving ECG (RR 1.39; 95% CI 1.21 to 1.59; 7 trials, *n* = 612).

#### 3.3.9. Angina Pectoris Immediate Effect

There was only one trial [25] which reported the angina pectoris immediate effect. 2 notoginsenoside pills were prescribed in this trial when angina pectoris happened. The criterion was defined as remarkably effective (angina was alleviated in 3 minutes); effective (angina was alleviated in 3–5 minutes); no effect (angina was alleviated in more than 5 minutes or need to add other medicines). The result indicated that notoginsenoside pill had similar effect compared with isosorbide dinitrate (RR 0.96; 95% CI 0.81 to 1.15; 1 trial, *n* = 80).

#### 3.3.10. Angina Pectoris Frequency

There were 4 studies [21, 26, 29, 33] reporting frequency of angina pectoris in the unit of times/week. Compared with no intervention on the basis of conventional therapy, *Panax notoginseng* preparation (Xuesaitong softy capsule) showed a reduction in angina pectoris frequency (MD −2.16; 95% CI −3.02 to −1.30; 4 trials, *n* = 572). Sensitivity analysis also indicated that *Panax notoginseng* preparation was effective in reducing angina pectoris frequency (MD −2.34; 95% CI −2.53 to −2.16; 2 trials, *n* = 292) [21, 29]. There was 1 trial [33] which reported the frequency of angina pectoris in the unit of times/day. The result indicated that *Panax notoginseng* (Xuesaitong softy capsule) could reduce angina pectoris frequency compared with no treatment on the basis of conventional therapy (MD −2.76; 95% CI −3.87 to −1.65; 1 trial, *n* = 28).

#### 3.3.11. Angina Pectoris Duration

There was 3 trials [21, 29, 33] reporting the duration of angina pectoris. The result showed that *Panax notoginseng* preparation (Xuesaitong softy capsule) significantly reduced angina pectoris duration comparing with no treatment on the basis of conventional therapy (MD −2.10; 95% CI −2.58 to −1.62; 3 trials, *n* = 472). However, there was significant statistical heterogeneity among these three trials (*I* ^2^ = 91%). Further sensitivity analysis also indicated the benefit of *Panax notoginseng* preparation (Xuesaitong softy capsule) in reducing angina pectoris frequency in hospitalized patients (MD −1.88; 95% CI −2.08 to −1.69; 2 trials, *n* = 292) [21, 29]. 

#### 3.3.12. Dosage of Nitroglycerol

There were 2 studies [21, 26] reporting dosage of nitroglycerol in the unit of mg/week. Compared with no intervention on the basis of conventional therapy, oral *Panax notoginseng* preparation (Xuesaitong softy capsule) showed a reduction of nitroglycerol dosage (MD −1.13; 95% CI −1.70 to −0.56; 2 trials, *n* = 212). There was 1 study [36] reporting dosage of nitroglycerol in the unit of mg/day, which showed *Panax notoginseng* preparation (Xuesaitong softy capsule) also reduced the nitroglycerol dosage significantly (MD −4.10; 95% CI −5.58 to −2.62; 1 trial, *n* = 28).

### 3.4. Publication Bias

**Figure 3:**
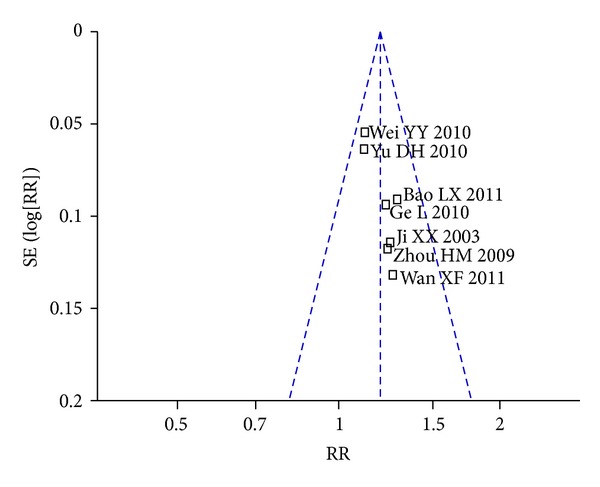
Funnel plot of comparison: conventional therapy and *Panax notoginseng* preparation versus conventional therapy, outcome: 3.3.7 Angina Pectoris Alleviation.

A funnel plot analysis of the 7 trials in comparison of *Panax notoginseng* preparation and conventional therapy versus conventional therapy on angina pectoris improvement was conducted and shown in Figure 3; there might be a publication bias in this review for small sample, negative report, and low quality of the included trials.

### 3.5. Adverse Events

There were 9 trials [21–25, 29–31, 33] reporting adverse events (Ads) (Table 4). 6 trials [21–24, 29, 33] indicated no Ads in the duration of treatment. 1 trial [25] reported reduction of blood pressure and increasement of heart rate (RR 0.03; 95% CI 0.00 to 0.543; 1 trial, *n* = 80); 1 trial [30] reported nausea (RR 3.0; 95% CI 0.13 to 70.16; 1 trial, *n* = 48); 1 trial [30] reported dizziness (RR 0.33; 95% CI 0.01 to 7.80; 1 trial, *n* = 48); 1 trial [30] reported vomit (RR 0.33; 95% CI 0.01 to 7.80; 1 trial, *n* = 48]); 1 trial [31] reported erythra (RR 3.00; 95% CI 0.13 to 71.92; 1 trial, *n* = 100). All Ads were not significantly different between the intervention group and the control group (Table 4).

## 4. Discussion

This systematic review included 17 RCTs and a total of 1747 participants. The review showed that, (1) comparing with no intervention on the basis of conventional therapy, oral *Panax notoginseng* showed no significant improvement for reducing the cardiovascular events, but it could relieve angina pectoris and related symptoms (including reducing the recurrence of angina pectoris, duration and frequency of angina pectoris, and dosage of nitroglycerol, as well as ECG changes); (2) oral *Panax notoginseng* showed similar immediate effect on angina pectoris compared with nitrate, but we could not make a significant conclusion from this equivalence due to small sample and low methodological quality trial; (3) The results also showed that oral *Panax notoginseng* was safe for CHD patients according to the information in hand, but it was too limited to make a conclusion for high risk bias and small sample in these trials. 

Oral *Panax notoginseng* preparations have been used widely for treating CHD in China. Most of the researchers paid more attention to their pharmacological mechanism. Yang et al. comprehensively collected the pharmacological action of *Panax notoginseng* and concluded that it could provide protective effects against cardiovascular diseases through many pharmacological mechanisms including improving myocardial microcirculation, reducing arrhythmia, regulating blood lipid, preventing atherosclerosis, lowering blood pressure, and antishock [37]. Du et al. summarized the experiments on *Panax notoginseng* for MI and concluded that *Panax notoginseng* could inhibit the inflammatory reaction and improve ischemia reperfusion injury in patients with MI [38]. Chan et al. concluded that Trilinolein purified from *Panax notoginseng* could provide protective effects against cardiovascular disease including reducing thrombogenicity and arrhythmia and increase erythrocyte deformability. It was also an antioxidant which could counteract free radical damage associated with atherogenesis and myocardial damage [39]. All these experiments provided us laboratory evidence on protective effect of *Panax notoginseng* for CHD. Although many clinical trials were conducted on effect of oral *Panax notoginseng* preparations for CHD, there was no critical appraisal for these up to now. There was still no enough evidence for clinicians to prescribe oral *Panax notoginseng* preparations in CHD patients. 

The impact of this review was to take a light on oral *Panax notoginseng* for CHD. Although it failed to prove the protective effect of *Panax notoginseng* on major cardiovascular events (cardiovascular mortality, MI incidence, and rehospitalization), it demonstrated that *Panax notoginseng* preparation might be recommended for improving symptoms of angina pectoris. 

However, before translating the conclusion of this review to clinical practitioners, we have to consider the following weaknesses in this review. (1) Firstly, the “randomization” was not clear in most of the trials for insufficient reporting of generation methods of the allocation sequence, allocation concealment. Most trials stated only that patients were randomly assigned. (2) Secondly, no trial used placebo in control group, most of trials did not introduce double blind in this review, and one trial introduced blinding of outcome assessment. Therefore, in nonplacebo-controlled and nondouble blind trials, placebo effects may add to the complexity of interpreting the conclusion. (3) Thirdly, most of the trials did not introduce the study plan, and attrition bias and selective reporting bias might exist in this conclusion. (4) Fourthly, funnel plot indicated that publication bias would exist in this review. The reasons we considered were as follows: we only selected trials published in Chinese and English trials published in other languages or originated from other countries might be omitted; we only identified unpublished studies from conference paper or academic thesis, and negative trials might not be reported and induce publication bias.

Although this review suggested some benefit of *Panax notoginseng* preparation for CHD, the recommendation should be discreet due to poor quality and high risk bias of these trials, further rigorously designed, and well reported RCTs are still needed to prove the effectiveness and safety of *Panax notoginseng* preparation for CHD.

## 5. Conclusion

In this systematic review, oral *Panax notoginseng* preparation did not show benefit on reducing major cardiovascular events and relapse (including cardiovascular death, MI incidence, incidence of intractable angina pectoris, and rehospitalization), although it was effective in alleviating angina pectoris (including the recurrence, frequency, and duration of angina pectoris, ECG presentation, and dosage of nitroglycerin) with low adverse reaction. However, the small sample size and potential bias of most trials influence the convincingness of this conclusion. Before recommending oral *Panax notoginseng* preparation as an alternative herbal medicine in CHD patients, more rigorous trials with high quality are needed to prove the benefit of oral *Panax notoginseng* preparation and provide high level of evidence.

## Figures and Tables

**Table 1:** Characteristics of trials.

Study ID	Type of CHD and syndrome	Members (I/C)	Age	Gender (M/F)	Interventions group	Control group	Product	Outcome evaluation
Du and Chen 2009 [21]	UA	56/56	58.8 ± 9.2	62/50	**C** + Xuesaitong softy capsule, 2 capsules, BID, 4 weeks	Conventional therapy (aspirin, *β* blocker agent, nitroglycerin, CCB, low molecular heparin 5–7 days, antihypertensive drugs and medicine used to treat 2 diabetes)	Xuesaitong softy capsule* (Shenghuo Pharmaceutical Holdings Yunnan kunming, China, Z19990022, containing PNS 60 mg/capsule)	Angina pectoris (extension, frequency, duration), dosage of nitroglycerin, Ads.

Ge and Zhao 2010 [22]	UA	48/48	I: 56; C: 54 (in average)	I: 22/26 V: 25/23	**C **+ Xuesaitong softy capsule, 2 capsules, BID, 4 weeks	Conventional therapy (aspirin, *β* blocker agent, nitroglycerin, CCB, low molecular heparin 5–7 days, antihypertensive drugs and medicine used to treat 2 diabetes)	Xuesaitong softy capsule* (Shenghuo Pharmaceutical Holdings Yunnan kunming China, Z19990022, containing PNS 60 mg/capsule)	Angina pectoris relievement, ECG, Ads.

Han and Chen 2008 [23]	PCI patients	30/30	I: (64.1 ± 10.8); C: (63.7 ± 11.7)	I: 23/7; C: 21/9	**C** + Xuesaitong softy capsule, 2 capsules, BID in the first 2 weeks, then 1 capsule, TID, 12 weeks	Conventional therapy (anticoagulant agent, antiplatelet agent, medicine for modifying blood lipid, antihypertensive drug and medicine used to treat 2 diabetes)	Xuesaitong softy capsule* (Shenghuo Pharmaceutical Holdings Yunnan kunming, China, Z19990022, containing PNS 60 mg/capsule)	Angina pectoris, rehospitalization

Ji and Zhang 2003 [24]	UA	30/90	I1: (69.0 ± 7.5); I2: (69.2 ± 6.0); I3: (68.5 ± 5.4); C: (68.7 ± 7.3)	I1: 20/10; I2: 18/12; I3: 21/9; C: 17/13	I1: **C** + coarse power 1 g, TID; I2: **C** + semi-micron power 1 g, TID I3: **C** + micron power 1 g, TID	Isosorbide Mononitrate 20 mg BID; Aspirin 75 mg, QD; Metoprolol 25 mg BID; DTZ 30 mg, TID or QID; Plendil 5 mg QD or BID or Acertil 4 mg, QD for hypertension; Nitroglycerol 0.5 mg subligual administration or nitroglycerol injection 10 mg, iv.	*Panax notoginseng* coarse power: WF-2000 pulverizer; *Panax notoginseng* micron power: BFM-6 pulverizer; *Panax notoginseng* semi-micron power: BFM-6 pulverizer and starch.	Efficacy of Angina pectoris, ECG, symptoms, Ads

Liu et al. 2008 [19]	UA and BSS	30/30	I: (64.6 ± 5.4); C: (63.6 ± 4.5)	Unclear	**C** + Xuesaitong softy capsule, 2 capsules, BID, 4 weeks	Conventional therapy (no detail)	Xuesaitong softy capsule (Yunnan weihe Pharmaceutical company, containing PNS 60 mg/capsule)	Syndrome, pulse, heart rate, heart rhythm, blood pressure, angina pectoris, ECG

Meng 2003 [25]	UA and SA	60/20	I: (61–78); C: (61–78)	I: 44/16 C: 16/4	PNS pill, 2 tablets, sublingual when angina pectoris attacked	Isosorbide dinitrate when angina pectoris attacked (5 mg/tables)	PNS pill, 2 tablet**, Sublingual	Duration of angina pectoris relievement, blood pressure, heart rate, and ECG after 2 hours of prescription.

Song et al. 2005 [26]	Unclear	50/50	I: (36–77), (61.21 ± 5.73); C: (38–74), (60.77 ± 5.61) in average	I: 31/19 C: 33/17	**C** + Xuesaitong softy capsule, 2 capsules, BID, 4 weeks	Conventional therapy (aspirin, *β* blocker agent, nitroglycerin, CCB, low molecular heparin 5–7 days, antihypertensive drugs and medicine used to treat diabetes)	Xuesaitong softy capsule* (Shenghuo Pharmaceutical Holdings Yunnan kunming, China)	Efficacy of angina pectoris, ECG, dosage of nitroglycerin

Wan 2011 [27]	UA	26/26	I: 65.7 in average; C: No report	I: 15/11 C: 13/13	**C** + Xuesaitong softy capsule, 2 capsules, BID, 4 weeks	Conventional therapy (aspirin, *β* blocker agent and et al.)	Xuesaitong softy capsule* (Shenghuo Pharmaceutical Holdings Yunnan kunming, China)	Efficacy of angina pectoris, ECG

Wang et al. 2009 [28]	UA	100/100	36–75	Unclear	**T1:C1 **+ Xuesaitong softy capsule, 2 capsules, BID, 30 days; **T2:C2 **+ trimetazidine + Xuesaitong softy capsule, 2 capsules, BID, 30 days;	C1: conventional therapy (ant platelet, Nitrates, CCB, *β* blocker agent, statin, trimetazidine); C2: Conventional therapy (ant platelet, Nitrates, CCB, *β* blocker agent, astatine)	Xuesaitong softy capsule	Efficacy of angina pectoris and cardiovascular events in 30 d followup.

Wei 2010 [29]	Unclear	90/90	60.4 ± 3.5	113/67	**C** + Xuesaitong softy capsule, 2 capsules, BID, 4 weeks	Conventional therapy (Nitrate, *β* blocker agent, CCB, low molecular heparin)	Xuesaitong softy capsule* (Shenghuo Pharmaceutical Holdings Yunnan kunming, China)	Angina pectoris, Ads, ECG

Yan 2005 [30]	Unclear	24/24	I: (48–67), 60 in average; C: (47–69), 62 in average	I: 13/11 C: 14/10	Isosorbide mononitrat 5 mg TID + *Panax notoginseng* power 6 g BID, 7 days	Isosorbide Mononitrate, 10 mg, TID	*Panax notoginseng* power 6 g BID	Efficacy of angina pectoris, ECG, ADs

Yu 2010 [31]	UA	50/50	I: (64.18 ± 12.13); C: (62.8 ± 10.8)	I: 29/21 C: 28/22	**C** + Xuesaitong softy capsule, 2 capsules, BID, 4 weeks	Conventional therapy (aspirin, *β* blocker agent, nitroglycerin, CCB and et al.)	Xuesaitong softy capsule* (Shenghuo Pharmaceutical Holdings Yunnan kunming, China)	Efficacy of angina pectoris, ECG, ADs, cardiovascular events

Zhou and Bai 2009 [32]	Unclear	43/43	65 ± 6	I: 32/11 C: 34/9	**C **+ Xuesaitong softy capsule, 2 capsules, TID, 4 weeks	Conventional therapy (nitrate, Metoprolol, aspirin, Nitroglycerin if necessary)	Xuesaitong softy capsule^Δ^ (Luotai, Kunming Pharmaceutical incorporated corporation)	Efficacy of angina pectoris, ECG

Kuang et al. 2011 [33]	UA	90/90	I: (56.3 ± 6.9); C: (57.1 ± 7.2)	I: 47/43 C: 46/44	**C **+ Xuesaitong softy capsule, 2 capsules, BID, 4 weeks	Conventional therapy (aspirin, *β* blocker agent, nitroglycerin, CCB, low molecular dextran, and others)	Xuesaitong softy capsule* (Shenghuo Pharmaceutical Holdings Yunnan kunming, China, containing PNS 60 mg/capsule)	Efficacy of angina pectoris, ECG, ADs

Bao 2011 [34]	SA, BSS	63/64	I: (52.3 in average); C: (51.6 in average)	I: 35/28 C: 37/27	**C **+ Sanqi guanxinning tablets (Z53020028), 2–4 tables, TID, 6 weeks	Conventional therapy (nitroglycerin, *β* blocker agent, and others)	Sanqi guanxinning tablets^ΔΔ^ (Z53020028)	Efficacy of angina pectoris

Zhao and Li 2012 [35]	SA	60/58	I: (57.4 ± 9.9, 42–70); C: (59.6 ± 9.7, 41–68)	I: 38/22 C: 40/18	**C **+ Xuesaitong softy capsule, 2 capsules, TID, 4 weeks	Conventional therapy (aspirin, J20080078, 100 mg) Qd, isosorbide mononitrate (H20030418, 60 mg Qd), *β* blocker (H32025391)	Xuesaitong softy capsule* (Shenghuo Pharmaceutical Holdings Yunnan kunming, China, Z19990022)	Efficacy of ECG

Yang 2012 [36]	Unclear	14/14	(67.3 ± 1.1), 51–78	19/9	**C **+ Xuesaitong softy capsule, 2 capsules BID in the first two weeks, 1 capsule BID in the later weeks	Conventional therapy (aspirin, *β* blocker agent, nitroglycerin, CCB, and others)	Xuesaitong softy capsule* (Shenghuo Pharmaceutical Holdings Yunnan kunming, China, containing PNS 60 mg/capsule)	Frequency of angina pectoris, dosage of nitroglycerin, frequency of premature ventricular contraction

BSS: blood stasis syndrome; PNS: *panax notoginseng* saponins; I: intervention group; C: control group; DTZ: dilthiazem; ECG: electrocardiogram; Ads: adverse event.

*Xuesaitong softy capsule produced by Shenghuo Pharmaceutical Holdings, Yunnan kunming, China (Z19990022) contains PNS 60 mg/capsule.

**There was no purity of PNS pill in this trial. According to the internet, PNS pill produced by Yunnan Weihe Pharmaceutical company contains PNS 50 mg/pill.

^Δ^Xuesaitong softy capsuleΔ (Luotai, Kunming Pharmaceutical incorporated corporation, China) contains PNS 100 mg/capsule.

^ΔΔ^Sanqi guanxinning tablets (Z53020028), there is no introduction in the paper about the composition and the purity. According to the internet, Sanqi guanxinning producted by Yunnan JinBuHuan (group) Co., ltd. pharmaceutical branch, containing 100 mg PNS/tablet.

**Table 2:** Analysis of cardiovascular events and angina pectoris.

Outcomes (comparisons)	Treatment group (*n*/*N*)	Control group (*n*/*N*)	RR	95% CI
(1) Cardiovascular mortality				
*Panax notoginseng* preparation and conventional therapy versus conventional therapy				
Yu 2010 [31]	1/50	2/50	0.50	[0.05, 5.34]
(2) Myocardial infarction incidence				
*Panax notoginseng* preparation and conventional therapy versus conventional therapy				
Wang et al. 2009 [28]	0/100	3/100	0.14	[0.01, 2.73]
Yu 2010 [31]	0/50	2/50	0.20	[0.01, 4.06]
	Overall all (FEM, *I* ^2^ = 0%)		0.17	[0.02, 1.37]
(3) Incidence of intractable angina pectoris				
*Panax notoginseng* preparation and conventional therapy versus conventional therapy				
Wang et al. 2009 [28]	6/100	11/100	0.55	[0.21, 1.42]
(4) Rehospitalization incidence for unstable angina				
*Panax notoginseng* preparation and conventional therapy versus conventional therapy				
Han and Chen 2008 [23]	1/30	3/30	0.33	[0.04, 3.03]
(5) Recurrence of angina pectoris				
*Panax notoginseng* preparation and conventional therapy versus conventional therapy				
Han and Chen 2008 [23]	5/30	13/30	0.38	[0.16, 0.94]
(6) Nitroglycerol decreasement				
*Panax notoginseng* preparation and conventional therapy versus conventional therapy				
Ji and Zhang 2003 [24]	19/30	65/120	1.17	[0.85, 1.61]
Song et al. 2005 [26]	26/50	14/50	1.86	[1.11, 3.12]
	Overall all (REM, *I* ^2^ = 59%)		1.41	[0.89, 2.24]
(7) Angina pectoris relievement				
*Panax notoginseng* preparation and conventional therapy versus conventional therapy				
Ge and Zhao 2010 [22]	44/48	36/48	1.22	[1.02, 1.47]
Ji and Zhang 2003 [24]	24/30	77/120	1.25	[1.00, 1.56]
Wan 2011 [27]	24/26	19/26	1.26	[0.98, 1.64]
Wei 2010 [29]	84/90	75/90	1.12	[1.01, 1.25]
Yu 2010 [31]	48/50	43/50	1.12	[0.98, 1.27]
Zhou and Bai 2009 [32]	37/43	30/43	1.23	[0.98, 1.55]
Bao 2011 [34]	57/63	45/64	1.29	[1.08, 1.54]
	Overall all (FEM, *I* ^2^ = 0%, *N* = 791)		1.20	[1.12, 1.28]
Subgroup analysis (excluded Ji and Zhang [24])	Overall (FEM, *I* ^2^ = 0%, *N* = 641)		1.19	[1.11, 1.27]
(8) Electrocardiogram improvement				
15.1 *Panax notoginseng* preparation and conventional therapy versus conventional therapy				
Ge and Zhao 2010 [22]	42/48	36/48	1.17	[0.96, 1.42]
Ji and Zhang 2003 [24]	67/86	19/29	1.19	[0.89, 1.58]
Liu et al. 2008 [19]	12/30	8/30	1.50	[0.72, 3.14]
Song et al. 2005 [26]	36/50	27/50	1.33	[0.98, 1.82]
Wan 2011 [27]	19/26	12/26	1.58	[0.98, 2.55]
Yu 2010 [31]	28/50	19/50	1.47	[0.96, 2.27]
Zhou and Bai 2009 [32]	35/43	27/43	1.30	[0.99, 1.70]
Zhao and Li 2012 [35]	24/60	12/58	1.93	[1.07, 3.49]
	Overall all (FEM, *I* ^2^ = 0%, *N* = 727)		1.35	[1.19, 1.53]
Subgroup analysis (excluded Ji and Zhang [24])	Overall (FEM, *I* ^2^ = 0%, *N* = 612)		1.39	[1.21, 1.59]
15.2 *Panax notoginseng* preparation and conventional therapy versus isosorbide dinitrate and conventional therapy				
Meng 2003 [25]	19/60	8/20	0.79	[0.41, 1.52]
(9) Angina pectoris immediate effect				
*Panax notoginseng* preparation and conventional therapy versus isosorbide dinitrate and conventional therapy				
Meng 2003 [25]	52/60	18/20	0.96	[0.81, 1.15]

FEM: fixed effects model; REM: random effects model; RR: relative risk; CI: credibility interval.

**Table 3:** Analysis of efficacy of angina pectoris.

Angina pectoris (comparison)	Intervention group		Control group		Weight (%)	MD	95% CI
	Mean	SD	Mean	SD			
(1) Angina pectoris frequency							
*Panax notoginseng* preparation and conventional therapy versus conventional therapy (times/week)							
Du and Chen 2009 [21]	3.24	0.61	5.63	0.92	33.6	−2.39	[−2.68, −2.10]
Kuang et al. 2011 [33]	3.53	0.61	6.83	1.92	14.1	−3.30	[−3.72, −2.88]
Song et al. 2005 [26]	0.75	0.79	1.36	1.31	32.4	−0.61	[−1.03, −0.19]
Wei 2010 [29]	4.27	0.87	6.58	0.75	34.0	−2.31	[−2.55, −2.07]
	Overall (REM, *I* ^2^ = 96%, *N* = 572)				**100**	**−2.16**	**[−3.02, −1.30]**
Sensitive analysis (excluded Song et al. 2005 [26] Kuang et al. [33])	Overall (FEM, *I* ^2^ = 0%, *N* = 292)					**−2.34**	**[−2.53, −2.16]**
*Panax notoginseng* preparation and conventional therapy versus conventional therapy (times/day)							
Yang 2012 [36]	1.22	0.97	3.98	1.89	28	−2.76	[−3.87, −1.65]
(2) Angina pectoris duration (minute/time)							
*Panax notoginseng* preparation and conventional therapy versus conventional therapy							
Du and Chen 2009 [21]	2.86	0.72	4.82	0.63	60.7	−1.96	[−2.21, −1.71]
Kuang et al. 2011 [33]	2.23	0.62	4.78	0.83	45.4	−2.55	[−2.76, −2.34]
Wei 2010 [29]	4.56	1.08	6.32	1.05	39.3	−1.76	[−2.07, −1.45]
	Overall (REM, *I* ^2^ = 91%, *N* = 472)				100	−2.10	[−2.58, −1.62]
Sensitive analysis (excluded Kuang et al., [33])	Overall (FEM, *I* ^2^ = 0%, *N* = 292)					**−1.88**	**[−2.08, −1.69]**
(3) Dosage of nitroglycerol							
*Panax notoginseng* preparation and conventional therapy versus conventional therapy (mg/week)							
Du and Chen 2009 [21]	2.94	2.26	4.26	1.94	53.0	−1.32	[−2.10, −0.54]
Song et al. 2005 [26]	2.95	2.25	3.87	1.97	47.0	−0.92	[−1.75, −0.09]
	Overall (FEM, *I* ^2^ = 0%, *N* = 212)				100	−1.13	[−1.70, −0.56]
*Panax notoginseng* preparation and conventional therapy versus conventional therapy (mg/day)							
Yang 2012 [36]	1.3	0.4	5.4	2.8	100	−4.10	[−5.58, −2.62]

FEM: fixed effects model; REM: random effects model; MD: mean difference; CI: credibility interval.

**Table 4:** Adverse Events.

Study ID	ADs
Du and Chen 2009 [21]	No abnormal changes appeared and no Ads was reported in the trial.
Ge and Zhao 2010 [22]	Blood regular test, urine regular test, and blood biochemistry test had no changes compared with the previous.
Han and Chen 2008 [23]	No serious Ads were reported in the trial; blood, urine, and stool routine tests, blood biochemistry test had no changes comparing with the previous.
Ji and Zhang 2003 [24]	Blood, urine, and stool routine tests, and blood biochemistry test had no changes compared with the previous. No Ads was reported in the trial.
Meng 2003 [25]	Reduction of blood pressure and increasement of heart rate: intervention group: 0/60; control group: 5/20. RR: 0.03. 95% CI: [0.00, 0.54].
Wei 2010 [29]	Blood, urine, and stool routine tests, and blood biochemistry test had no changes compared with the previous. No Ads was reported in the trial.
Yan 2005 [30]	Nausea: intervention group (1/24), control group (0/24), RR: 3.0, 95% CI: [0.13, 70.16]. Dizziness: intervention group (0/24), control group (1/24), RR: 0.33, 95% CI: [0.01, 7.80]. Vomit: intervention group (0/24), control group (1/24), RR: 0.33, 95% CI: [0.01, 7.80].
Yu 2010 [31]	Erythra: intervention group (1/50), control group: (0/50). RR: 3.00; 95% CI: [0.13, 71.92].
Kuang et al. 2011 [33]	No abnormal changes appeared and no Ads was reported in the trial.

Note: ADs: Adverse Events.
